# Lung ultrasonography as a marker of pulmonary edema in cardiac surgery patients: visual versus quantitative evaluation

**DOI:** 10.1186/cc14301

**Published:** 2015-03-16

**Authors:** F Corradi, C Brusasco, T Manca, F Nicolini, F Nicosia, T Gherli, V Brusasco, A Vezzani

**Affiliations:** 1 Ente Ospedaliero Ospedali Galliera Genova, Italy; 2 Università degli Studi di Genova, Italy; 3 Azienda Ospedaliero-Universitaria di Parma, Italy

## Introduction

Lung ultrasonography (LUS) has been used for noninvasive detection of pulmonary edema. LUS visual scores (V-LUS) based on B-lines are poorly correlated with either pulmonary capillary wedge pressure (PCWP) or extravascular lung water (EVLW). A new quantitative LUS analysis (Q-LUS) has been recently proposed [1,2]. The aim of the study was to investigate whether Q-LUS is better correlated with PCWP and EVLW than V-LUS, and to what extent positive end-expiratory pressure (PEEP) affects the assessment of pulmonary edema by Q-LUS and V-LUS.

## Methods

Thirty-nine patients mechanically ventilated with PEEP of 5 cmH_2_O (*n *= 47) or 10 cmH_2_O (*n *= 30) and PCWP (*n *= 77) or EVLW (*n *= 38) monitored were studied.

## Results

PCWP was significantly and strongly correlated with Q-LUS Grey Unit value (*r*^2 ^= 0.64) but weakly with V-LUS B-line score (*r*^2 ^= 0.19). EVLW was significantly and strongly correlated with QLUS Grey Unit mean value (*r*^2 ^= 0.65) more than with V-LUS B-line score (*r*^2 ^= 0.42). Q-LUS showed a better diagnostic accuracy than V-LUS for the detection of PCWP >15 mmHg or EVLW >10 ml/kg. With a PEEP of 5 cmH_2_O, the correlations with PCWP or EVLW were stronger with Q-LUS than V-LUS. With a PEEP of 10 cmH_2_O, the correlations with PCWP or EVLW were still significant for Q-LUS but insignificant for V-LUS. Intraobserver repeatability and interobserver reproducibility were much better for Q-LUS than V-LUS.

## Conclusion

Both V-LUS and Q-LUS are acceptable indicators of pulmonary edema in patients mechanically ventilated with low PEEP but at high PEEP only Q-LUS provides data that are significantly correlated with. Computer-aided Q-LUS has the advantages of being not only independent of operator perception but also of PEEP.
